# Protecting Stem Cell Derived Pancreatic Beta-Like Cells From Diabetogenic T Cell Recognition

**DOI:** 10.3389/fendo.2021.707881

**Published:** 2021-07-09

**Authors:** Roberto Castro-Gutierrez, Aimon Alkanani, Clayton E. Mathews, Aaron Michels, Holger A. Russ

**Affiliations:** ^1^ Barbara-Davis Center for Diabetes, University of Colorado Anschutz, Aurora, CO, United States; ^2^ Department of Pathology, Immunology, and Laboratory Medicine, College of Medicine, University of Florida, Gainesville, FL, United States

**Keywords:** autoimmune type 1 diabetes, stem cell derived pancreatic insulin producing beta-like cells, beta- immune cell interface, PD-L1 and HLA class I, beta cell replacement therapy, genome engineering, human pluripotent stem cells, direct differentiation

## Abstract

Type 1 diabetes results from an autoimmune attack directed at pancreatic beta cells predominantly mediated by T cells. Transplantation of stem cell derived beta-like cells (sBC) have been shown to rescue diabetes in preclinical animal models. However, how sBC will respond to an inflammatory environment with diabetogenic T cells in a strict human setting has not been determined. This is due to the lack of model systems that closely recapitulates human T1D. Here, we present a reliable *in vitro* assay to measure autologous CD8 T cell stimulation against sBC in a human setting. Our data shows that upon pro-inflammatory cytokine exposure, sBC upregulate Human Leukocyte Antigen (HLA) class I molecules which allows for their recognition by diabetogenic CD8 T cells. To protect sBC from this immune recognition, we utilized genome engineering to delete surface expression of HLA class I molecules and to integrate an inducible overexpression system for the immune checkpoint inhibitor Programmed Death Ligand 1 (PD-L1). Genetically engineered sBC that lack HLA surface expression or overexpress PD-L1 showed reduced stimulation of diabetogenic CD8 T cells when compared to unmodified cells. Here, we present evidence that manipulation of HLA class I and PD-L1 receptors on sBC can provide protection from diabetes-specific immune recognition in a human setting.

## Introduction

Type 1 diabetes mellitus (T1D) results from the autoimmune-mediated destruction of insulin producing beta cells in the pancreas (1). This specific beta cell depletion results in life threatening hyperglycemia if left untreated. Exogenous administration of insulin is necessary to treat the metabolic disturbances in T1D. However, management of the disease with insulin therapy remains challenging with risk for acute hypoglycemia and severe long-term complications. Despite the advancements in T1D technology including subcutaneous insulin pumps, continuous glucose monitors, and hybrid closed loop insulin delivery systems (2), a practical cure is still lacking.

Replacing lost beta cells by transplantation of cadaveric donor islets, which contain beta cells, along with simultaneous immune suppression has been proposed as a cure for T1D (3). Islet transplantation into T1D patients results in insulin independence for an average of 34 months (4), demonstrating a proof of principle that beta cell transplantation could indeed represent a practical cure for T1D patients. However, important caveats still remain: insufficient donor tissue availability, donor incompatibility, and remaining autoimmunity (5). To address the shortage of donor tissue for transplantation, research efforts have focused on using direct differentiation of human pluripotent stem cells (hPSC) into pancreatic and insulin producing, stem cell derived beta-like cells (sBC) to effectively create an abundant source of functional beta cells (6–10). At present it is still largely unknown how sBC respond to a human diabetogenic environment. This is critically important knowledge that is needed to protect sBC from immune rejection and autoimmune-related injury prior to successful transplantation into T1D patients.

Transplantation of macroencapsulated sBC progenitor cells into animal models is a viable approach to confer protection from the host immune system, but cell survival and function has been disappointing. Indeed, scar tissue formation, foreign body reactions, and poor vascularization are critical concerns for this technology (11). Microencapsulation of sBC using alginate derivatives allows for a better exchange of nutrient and gases which are important for sBC function, thus providing an attractive approach for cell replacement therapy (12). However, transplantation of naked sBC, without the need for cell encapsulation, would provide distinct benefits. Naked sBC grafts would be readily vascularized, improving survival and function, while mitigating foreign body reactions. On the other hand, naked sBC are more likely vulnerable to an immediate inflammatory environment and an immune attack without a protective layer provided by encapsulation approaches. Thus, alternative strategies need to be developed to address the challenges associated with cell replacement therapy using sBC for patients suffering from diabetes.

Human beta cells upregulate multiple receptors in response to an inflammatory environment in order to interact with the immune system. It is known that human beta cells express human leukocyte antigen (HLA) class I molecules which are implicated in the pathogenesis of T1D by presentation of diabetogenic antigens to CD8 T cells (13, 14). Self-reactive cytotoxic CD8 T cells with T cell receptors (TCRs) specific for beta cell antigens have been identified in T1D patients (15). However, not much is known regarding how sBC will react to these self-reactive, diabetogenic CD8 T cells that are already in circulation in T1D patients. This gap in knowledge is primary due to lack of a reliable assay to measure T cell responses towards sBC in a strictly human autologous setting.

Furthermore, cadaveric beta cells can upregulate the immune checkpoint inhibitor Programmed Death-Ligand 1 (PD-L1) receptor in response to pro-inflammatory cytokines (16–18) as a protecting mechanism. Interestingly, anti-PD-1 and anti-PD-L1 cancer therapy has resulted in spontaneous development of autoimmune diabetes in a subset of patients (19–21). Additionally, a recent study indicates that sBC overexpressing PD-L1 survive longer after transplantation into immune competent mice (22), implicating an important role for PD-L1 in providing peripheral tolerance to beta cells and preventing autoimmune diabetes. However, this study was limited by the use of a xenogeneic model.

Here we established a reproducible, well-defined assay system to measure T cell stimulation *in vitro* in order to study human sBC and T-cell interactions. We confirmed that sBC upregulate HLA class I molecules similar to human islet beta cells when exposed to pro-inflammatory cytokines. We further demonstrated that sBC are able to stimulate diabetogenic human T cells reactive to the self-antigen preproinsulin (PPI) in an HLA-peptide-TCR dependent manner. Then we sought to evaluate the hypothesis that sBC lacking HLA class I molecules and overexpressing PD-L1 would not be recognized by diabetogenic CD8 T cells when exposed to inflammatory cytokines. Using this novel system we provided direct evidence that sBC stimulate human diabetogenic CD8 T cells in an HLA-dependent manner and that PD-L1 overexpression can effectively reduce this stimulation.

## Materials and Methods

### hPSC Culture and Differentiation of Stem Cell Derived Beta-Like Cells (sBC)

Undifferentiated human pluripotent stem (hPSC) Mel1^INS-GFP^ reporter cells (23) were maintained on hES qualified Matrigel (Corning #354277) in mTeSR+ media (STEMCELL Technologies #05826). Differentiation to stem cell derived beta like cells (sBC) was carried out in suspension‐based, bioreactor magnetic stirring system (Reprocell #ABBWVS03A-6, #ABBWVDW-1013, #ABBWBP03N0S-6) as follows. Confluent hPSC cultures were dissociated into single‐cell suspension by incubation with TrypLE (Gibco #12-604-021) for 8 min at 37C. Detached cells were quenched with mTESR+ media. Cells were then counted using a MoxiGo II cell counter (Orflow), followed by seeding 0.5 × 10^6^ cells/ml in mTeSR+ media supplemented with 10uM ROCK inhibitor (Y-27632, R&D Systems #1254-50). Bioreactors were placed on a magnetic stirring system set at 60rpm in a cell culture incubator with 5% CO_2_ to induce sphere formation for 48 hours. To induce definitive endoderm differentiation, spheres were collected in a 50ml Falcon tube, allowed to settle by gravity, washed once with RPMI (Gibco #11-875-093) + 0.2% FBS, and re‐suspended in d1 media [RPMI containing 0.2% FBS, 1:5,000 ITS (Gibco #41400-045), 100ng/ml Activin A (R&D Systems #338-AC-01M), and 3uM CHIR99021 (STEMCELL Technologies #72054)]. Differentiation media was changed daily by letting spheres settle by gravity for 3-10min. Most supernatant was removed by aspiration; fresh media was added, and bioreactors were placed back on stirrer system. sBC differentiation was based on published protocol (Russ et al, 2015) with modifications as outlined below. Differentiation medias are as: day 2-3: RPMI containing 0.2% FBS, 1:2,000 ITS, and 100ng/ml Activin A; d4-5: RPMI containing 2% FBS, 1:1,000 ITS, and 50ng/ml KGF (Prepotech #100-19-1MG); d6: DMEM with 4.5g/L D-glucose (Gibco #11960-044) containing 1:100 SM1 (STEMCELL Technologies #5711), 1:100 NEAA (Gibco #11140-050), 1mM Sodium Pyruvate (Gibco #11360-070), 1:100 GlutaMAX (Gibco #35050-061), 3nM TTNPB, (R&D Systems #0761), 250nM Sant-1 (R&D Systems #1974), 250nM LDN (STEMCELL Technologies #72149), 30nM PMA (Sigma Aldrich #P1585-1MG), 50ug/ml 2-phospho-L-ascorbic acid trisodium salt (VitC) (Sigma #49752-10G); d7: DMEM containing 1:100 SM1, 1:100 NEAA, 1mM Sodium Pyruvate, 1:100 GlutaMAX, 3nM TTNPB, and 50ug/ml VitC; d8-9: DMEM containing 1:100 SM1, 1:100 NEAA, 1mM Sodium Pyruvate, 1:100 GlutaMAX, 100ng/ml EGF (R&D Systems #236-EG-01M), 50ng/ml KGF, and 50ug/ml VitC; d10-16: DMEM containing 2% fraction V BSA, 1:100 NEAA, 1mM Sodium Pyruvate, 1:100 GlutaMAX, 1:100 ITS, 10ug/ml Heparin (Sigma #H3149-250KU), 2mM N-Acetyl-L-cysteine (Cysteine) (Sigma #A9165-25G), 10uM Zinc sulfate heptahydrate (Zinc) (Sigma #Z0251-100g), 1x BME, 10uM Alk5i II RepSox (R&D Systems #3742/50), 1uM 3,3’,5-Triiodo-L-thyronine sodium salt (T3) (Sigma #T6397), 0.5uM LDN, 1uM Gamma Secretase Inhibitor XX (XXi) (AsisChem #ASIS-0149) and 1:250 1M NaOH to adjust pH to ~7.4; d17-23: CMRL (Gibco #11530-037) containing 1% BSA, 1:100 NEAA, 1mM Sodium Pyruvate, 1:100 GlutaMAX, 10ug/ml Heparin, 2mM Cysteine, 10uM Zinc, 1x BME, 10uM Alk5i II RepSox, 1uM T3, 50ug/ml VitC, and 1:250 NaOH to adjust pH to ~7.4. All medias also contained 1x PenStrep.

### Human Islet Culture

Human islets were cultured in CMRL media containing 10% FBS, 1X Pen/Strep, 1X BME.

**Table:** 

Unos ID/Batch Type	Purity	Institute	BMI	Age
R290	80%	University of Alberta	35.8	74
R299	75%	University of Alberta	25.4	44
RRID : SAMN11000549	85%	The Scharp-Lacy Research Institute	32.9	47

### Immunofluorescence

sBC and human islet clusters were dissociated for (i) cytospin studies or (ii) embedded for cryo-sectioning. (i) huma islets and sBC clusters were washed with PBS and incubated with 0.05% Trypsin with EDTA at 37 C for 12 min to create a single cell suspension. Cells were quenched with 2% FBS in PBS and filtered using a cell strainer. Cells were cytospun into glass slides and fixed for 15 min at room temperature with 4% paraformaldehyde. (ii) sBC clusters or human islets were fixed with 4% paraformaldehyde then washed twice with PBS. Fixed clusters for cryo-sectioning were incubated overnight in 30% sucrose (Sigma #S0389) before embedding in tissue-tek OCT (Sakura #4583) and storing at -80C overnight. OCT blocks containing fixed clusters were cryo-sectioned (10um thickness) and transferred to glass slides. Blocking and staining of cryo-sections proceeded as per whole mount staining protocol above. Antibody dilutions were prepared as indicated in **Table 1**. Images were acquired using confocal microscopy (Carl Zeiss LSM 800) using 10X, and 20X objectives. Where appropriate, cells were counted using Image J software.

**Table 1:** Reagents.

	Dilution	Company	Catalog #
**1ry Antibodies**			
rat anti-CPEP	1:400	Hybridoma/DSHB	GN-ID4
ms anti-PD-L1	1:100 IF/1:1000 WB	Cell signaling	41726S
rb anti-OCT4	1:50	Santa Cruz	sc-9081
ms anti-NKX6.1	1:200	Hybridoma/DSHB	F55A10
gt anti-PDX1	1:200	R&D	AF2419
rb anti-GAPDH	1:1000 WB	Abcam	ab9485
**Conjugated Antibodies**			
ms anti-TRA160 AF647	1:100	Biolegend	330606
ms anti-SOX2 AF594	1:100	Biolegend	656106
ms anti-FOXA2 PE	1:200	BD Biosciences	561589
ms anti-SOX17 AF488	1:200	BD Biosciences	562205
mIgG2a k Iso ctrl	1:100	Biolegend	400252 B217621
ms anti-HLA-ABC PerCP Cy5.5	1:100	Biolegend	311420 B227388
ms anti-PD-L1 PE-Cy7	1:100	Invitrogen	25-5983-41
ms anti-PDX1 PE	1:25	BD Biosciences	562161
ms anti-HLA-A*0201 BV421	1:100	BD Biosciences	740082
anti-PD1 PE	1:100	BD Biosciences	551892
**2ry Antibodies**			
anti-ms AF555	1:500	Thermo Fisher	A-31570
anti-rb AF555	1:500	Thermo Fisher	A-31572
anti-ms AF647	1:500	Thermo Fisher	A-31571
anti-rat AF488	1:500	Thermo Fisher	A-21208
anti-rb AF488	1:500	Thermo Fisher	R37118
anti-gt AF647	1:500	Thermo Fisher	A-21447
anti-rb HRP	1:1000	Invitrogen	31460
anti-ms HRP	1:1000	Invitrogen	31430
**RNA Probes**			
SOX2	1:20	BioRad	qHsaCEP0039595
NANOG	1:20	BioRad	qHsaCEP0050656
PDL1	1:20	BioRad	Hs00204257_m1
GAPDH	1:60	BioRad	qHsaCEP0041396
ACTINB	1:60	BioRad	Hs99999903_m1
HLA-A	1:20	BioRad	Hs01058806_g1
HLA-B	1:20	BioRad	Hs00818803_g1
HLA-C	1:20	BioRad	Hs00740298_g1
HLA-E	1:20	BioRad	Hs03045171_m1
HLA-F	1:20	BioRad	Hs01587840_m1
HLA-G	1:20	BioRad	Hs00365950_g1

### Flow Cytometry

hPSC and differentiating clusters were collected and dissociated as outlined above. Single cells were filtered through cell strainer into FACS 5ml tubes and incubated for 30 min on ice for surface markers or overnight at 4C for intracellular markers. After incubation, the cells were washed and strained again through cell strainer and resuspended in FACS buffer for analyses on CYTEK Aurora. Analysis and graphs were made using FloJo software v10.6.2.

### RT-qPCR

Total RNA was isolated using micro-RNeasy kit (Qiagen #74104) and reverse transcribed using the iSCRIPT cDNA kit (BioRad #1708891) as per manufacturer’s instructions. qPCR analysis was performed on BioRad CFX96 Real Time System using TaqMan probes described in **Table 1**.

### Western Blotting

Total protein was extracted from ~4x10^6^ cell using RIPA lysis buffer. Lysis buffer was supplemented with EDTA-free protease inhibitor (4693159001) and phosphatase inhibitor (Roche, 04906837001) cocktails. Lysates were resolved on SDS–PAGE, transferred to a methanol-activated PVDF membrane (BioRad, 1620177), blocked in 5% milk for 30 min, followed by incubation with the indicated primary antibodies (**Table 1**) overnight at 4C. After washing, membranes were then incubated for 1hr at room temperature with secondary antibody conjugated to horseradish peroxidase (HRP) as indicated in **Table 1**. After incubation with Clarity Western ECL Substrate (BioRad, 1705061), bands were detected with Azure Biosystems Western Blotting imager.

### CRIPSR-Cas9 and TALEN Mediated Genome Engineering

hPSC Mel1^INS-GFP^ cells were dissociated into single cells using TrypLE incubation at 37C for 8 min. Cells were then quenched with mTeSR+ media and counted using MoxiGo II cell counter. 2x10^6^ cells were transferred into microcentrifuge tubes and washed twice with PBS. Washed cells were then prepared for nucleofection of TALEN mediated knock-in (KI) of a Tet-On PD-L1 inducible system (i) or a CRIPSR-Cas9 mediated B2M gene knock-out (ii). Cells were nucleofected in P3 buffer following the Amaxa P3 Primary cell 4D-Nucleofector kit protocol (V4XP-3024) using the CB-150 program. (i) AAVS1-TALEN-L and AAVS1-TALEN-R (gift from Danwei Huangfu, Addgene plasmid # 59025; http://n2t.net/addgene:59025; RRID: Addgene 59025) as well as a *Tet-On* inducible PD-L1 overexpression plasmid (generated in house) were nucleofected into hPSC Mel1^INS-GFP^ cells. Nucleofected cells were then plated in 10cm plates with 10uM ROCK inhibitor. After 48hr of plating, puromycin selection (0.5ug/ml) was performed for 48 hr then removed for 48hr followed by neomycin selection (50ug/ml) for 6 days. Remaining colonies were picked and amplified for further characterization. (ii) guide RNAs (gRNA) targeting exon 1 and exon 2 of the B2M gene were generated in house and nucleofected along with CRISPR-Cas9. 24hr after plating, cells were selected for 48hr with puromycin (0.5ug/ml). Single colonies were picked and amplified for further characterization. Genomic DNA was extracted from targeted colonies and PCR analysis for TALEN KI and B2M KO was performed to identify potential modified clones. Further Sanger sequencing of PCR products confirm B2M mutations in the genome of hPSC Mel1^INS-GFP^ cells.

### T Cell Stimulation Assay

sBC clusters were washed with PBS and incubated with 0.05% Trypsin with EDTA at 37C for 12 min to create single cell suspension and quenched with 2% FBS in PBS. Cells were counted and plated into Matrigel coated 96 well plates at different densities for T cell stimulation assays or in 24 well plates for flow cytometry characterization. After 24hr sBC received an acute pro-inflammatory cytokine treatment (48hr IFN-*γ*, IL-1β, TNF-α) with or without 2µg/ml of Doxycycline hyclate (DOX; Sigma-Aldrich #D9891-1G). Cells were then washed twice with PBS and incubated with PPI: 15-24 (ALWGPDPAAA) at 10µg/ml or insulin B 13-23 (EALYLVCGERG) peptide at 100µg/ml for 4 hours. The insulin peptides used for these stimulation assays were obtained from Genemed Synthesis Inc. at > 95% purity and dissolved in PBS at a neutral pH. 1x10^5^ 5KC-1E6 T cells (TCR transductant responding to PPI: 15-24 presented by HLA-A*02:01) or 5KC-clone 5 (TCR transductant responding to insulin B 13-23 presented by HLA-DQ8) were co-culture with sBC overnight. 5KC T cells cultured without sBC or peptide were used as a negative control, and those treated with anti-CD3 monoclonal antibody (eBioscience, clone 2C11) at a concentration of 10µg/ml as positive control for each experiment. Murine IL-2 secreted by the 5KC cells was measured in the culture supernatant using a highly sensitive ELISA (V-PLEX IL-2 kit, Meso Scale Diagnostics, LLC) followed by detection on the MESO QuickPlex SQ120 instrument.

### Statistical Analysis

All statistical analyses were performed in GraphPad Prism (Version 9.0.0). Student t test was used for samples with 2 groups. For multiple group analyses One- or Two-way ANOVA’s was used with suggested multiple comparison tests. Paired test was employed with samples within the same bioreactor differentiation experiment.

### Data and Resource Availability

No datasets were generated or analyzed during the current study.

## Results

### Human Islets and sBC Differentially Express HLA and PD-L1 Molecules Upon Cytokine Exposure

**Figure 1:**
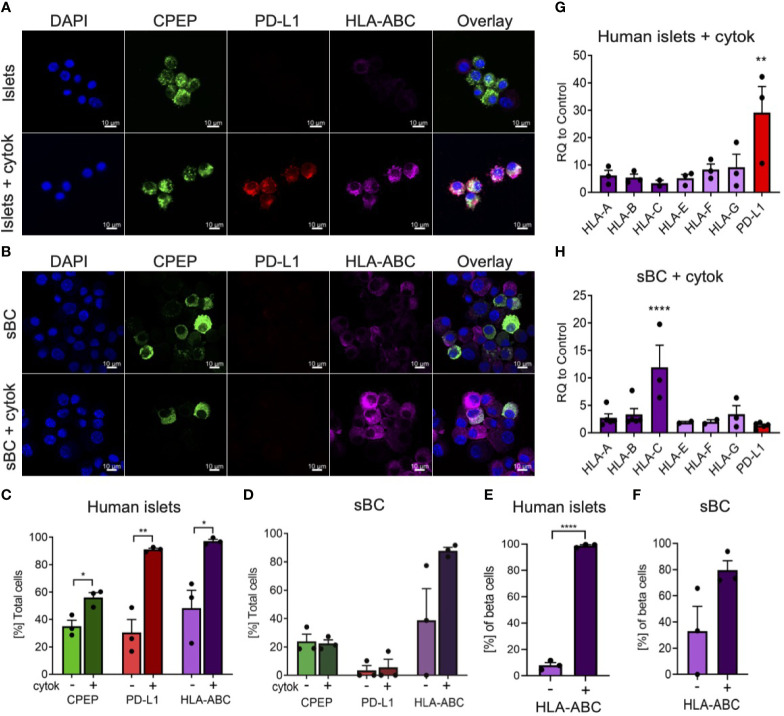
Human islets and sBC differentially upregulate HLA class I and PD-L1 molecules upon inflammatory conditions. **(A, B)** Representative immunofluorescence images of control and cytokine treated human islets **(A)** or sBC **(B)** for 6 days. Samples were stained for DAPI (blue), CPEP (green), HLA-ABC (purple), and PD-L1 (red). Scale bar represents 10um. **(C–F)** Quantification of percentage CPEP, HLA-ABC, or PD-L1 positive cells over total cell count **(C, D)** or over total CPEP cell count **(E, F)** of control and cytokine treated human islets **(C, E)** or sBC (D, F). Results are shown as mean +/- SEM of n=3 independent donors. Unpaired student t test was performed with *p ≤ 0.05, **p ≤ 0.01, and ****p ≤ 0.0001. **(G, H)** qPCR gene expression analysis of multiple HLA class I isoforms and PD-L1 after 6 days of cytokine treatment in human islets **(G)** or sBC **(H)**. Values are normalized to ACTB and relative to control. Results are shown as mean +/- SEM of n=3 independent donors and differentiation experiments. Ordinary One-Way Anova with Dunnett’s multiple test with **p ≤ 0.01, and ****p ≤ 0.0001.

During inflammation, beta cells are known to upregulate HLA class I and PD-L1 molecules in response to pro-inflammatory cytokines (13, 14, 16–18). These molecules are expressed on the cell surface of beta cells and help regulate immune cell responses. In order to determine if sBC exposed to inflammatory conditions exhibit a respond akin to bona fide beta cells, hPSC Mel1^INS-GFP^ cells were differentiated into sBC using a directed differentiation protocol. Mel1^INS-GFP^ cells contain a green fluorescence protein (GFP) reporter under the control of the endogenous insulin promoter that allows live monitoring of sBC generation (23). sBC and human islets in suspension culture were subjected to a 6-day cytokine treatment of IFN-*γ* 10ng/ml, IL-1β 0.5ng/ml and TNF-α 1ng/ml. Immunofluorescence staining of dissociated sBC or of sBC cluster sections indicates that sBC and human islets upregulate HLA-ABC molecules upon chronic cytokine treatment (**Figures 1A, B** and **Supplementary Figure 1A**). However, we do note that untreated sBCs exhibit considerable variability of HLA-ABC protein expression. Quantification shows that ~90% of treated sBC and human islets express HLA-ABC when exposed to pro-inflammatory cytokines (**Figures 1C, D**). Beta cell specific quantification demonstrates similar results for beta cells (**Figures 1E, F**). pan-HLA-ABC antibody used in immunofluorescence experiments cannot distinguish expression between different HLA class I isotypes. For this reason, quantitative PCR for specific isotypes was performed. This analysis shows a comparable upregulation of all HLA class I molecules tested in human islets (**Figure 1G**). Interestingly, sBC preferentially upregulated HLA-C upon pro-inflammatory cytokine treatment, while other HLA molecules were mostly unchanged compared to control conditions (**Figure 1H**). As preferential HLA-C expression has been previously observed in fetal tissues (24, 25), this could be an indicator that sBC are more closely aligned to fetal rather than adult beta cells.

Next, we evaluated the expression changes of PD-L1 upon cytokine treatment in human islets and sBC. PD-L1 is an important immune checkpoint inhibitor that can dampen immune cell activation in peripheral tissues (18). Thus, expression of PD-L1 in sBC could be important for regulating immune recognition and destruction by activated lymphocytes. Immunofluorescence staining and gene expression analysis confirmed a robust PD-L1 upregulation in human islets but not sBC after cytokine treatment (**Figures 1C, D, G, H**).

Taken together, this data indicates that induction of HLA class I and PD-L1 expression in respond to cytokine treatment differs between sBC and human islets *in vitro*. This data also shows that human islets and sBC react differently to an inflammatory environment.

### Integration of a PD-L1 Inducible System and HLA Class I Knock-Out in hPSC

**Figure 2:**
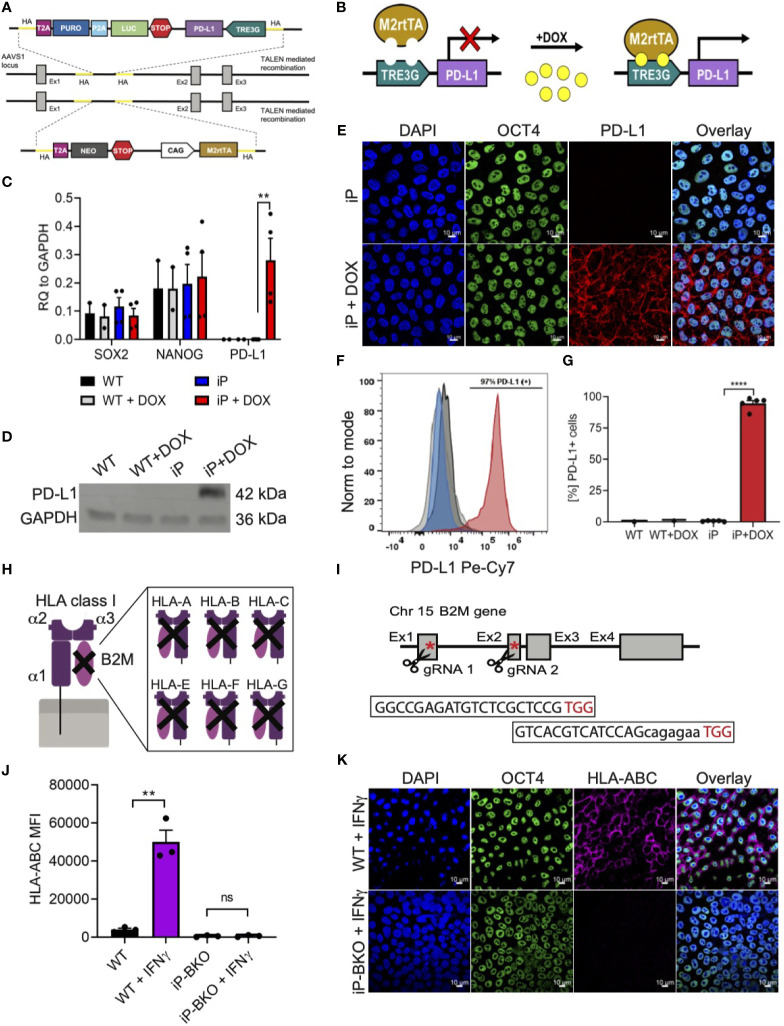
Integration of immune protective gene editing in hPSC using genome engineering. **(A, B)** Schematic of TALEN-mediated recombination strategy for the integration **(A)** and mechanism of action **(B)** of a Tet-On DOX inducible PD-L1 expression system between exon1 and 2 of the AAVS1 locus in the hPSC Mel1^INS-GFP^ line. **(C)** qPCR analysis of control hPSC (WT) and inducible PD-L1 hPSC (iP) cells with and without DOX treatment (2ug/ml for 48hr). Pluripotency markers SOX2 and NANOG were also analyzed. Values are normalized to GAPDH and shown as mean +/- SEM of n = 2 for WT and n = 4 for iP samples. 2way-Anova with Tukey’s multiple comparison test was performed with **p ≤ 0.01. **(D)** Immunoblotting for PD-L1 and loading control GAPDH proteins in WT and iP cells with and without DOX treatment. Representative immunoblot of 3 independent repeats. Complete blot is presented in **Supplementary Figure 2B**. **(E)** Representative immunofluorescence images of iP cells with and without DOX treatment. Sample were stain for DAPI (blue), OCT4 (green), and PD-L1 (red). Representative image of 4 independent experiments. Scale bar represents 10um. **(F, G)** Representative flow cytometry histogram **(F)** and quantification **(G)** for the detection of surface PD-L1 protein on WT (black), WT + DOX (light gray), iP (blue), and iP + DOX (red) cells. Results from n = 5 for iP and n = 1 for WT are shown as mean +/- SEM. Unpaired student’s t test was performed with ****p ≤ 0.0001. **(H, I)** Structural schematic of HLA class I complex with membrane bound α1 region, peptide binding grove composed of α2 and α3 regions and the invariable B2M region **(H)**. Inhibition of surface expression of all HLA class I isoforms is achieved by deletion of functional B2M using CRIPSR-Cas9 technology. Sequence for the two gRNAs specific for exons 1 and 2 with corresponding PAM sequences (in red) and location in relation to the B2M gene is shown with red asterisks **(I)**. **(J)** Flow cytometry quantification of surface HLA-ABC expression on WT and iP cells with B2M KO (iP-BKO) after IFN-*γ* treatment (100ng/ml for 48hr). MFI values are presented as mean +/- SEM for 3 independent experiments. Unpaired student’s t test was performed with **p ≤ 0.01; ns, not significant. **(K)** Representative immunofluorescence images of iP-BKO cells after IFN-*γ* stimulation. Samples were stain for DAPI (blue), OCT4 (green), and HLA-ABC (purple). Scale bar represents 10um.

In order to engineer sBC that can potentially withstand an (auto) immune attack, a Tet-On doxycycline (DOX) inducible PD-L1 expression system (iP) was integrated site specifically into the AAVS1 locus of hPSC using Transcription activator-like effector nucleases (TALEN) technology (**Figures 2A, B**) (26). To create a clonal line, double selection for puromycin and neomycin was applied to Mel1^INS-GFP^ cells that were nucleofected with two targeting and two TALEN plasmids followed by clonal selection after 11-13 days. Site specific integration of both targeting constructs was confirmed by genomic PCR analysis using specific primers for detecting neomycin and puromycin resistance genes in the AAVS1 loci (**Supplementary Figure 2A**). Functional testing of established iP line by DOX treatment for 48 hours revealed robust induction of PD-L1 mRNA expression while untreated controls displayed minimal leakiness of the inducible expression system (**Figure 2C**). Expression of key pluripotency markers SRY-Box Transcription Factor 2 (SOX2) and the Homeobox Transcription Factor Nanog (NANOG) were not changed in iP cells compared to unmodified wildtype (WT) cells (**Figure 2C**). Confirming the qPCR analysis, PD-L1 protein expression was readily detected by western blot at in DOX treated iP cells but not WT cells (**Figure 2D** and **Supplementary Figure 2B**). Immunofluorescence and surface flow cytometric analysis further demonstrated that PD-L1 protein was localized to the surface cell membrane in ~95% (+/- 4.4 STDV) of cells treated with DOX (**Figures 2E–G**). This data shows the generation of a clonal and functional Tet-On inducible PD-L1 expression system integrated into the AAVS1 loci of Mel1^INS-GFP^ hPSC.

HLA class I molecules play an important role interfacing beta cells with cytotoxic CD8 T cells. Removing HLA class I surface expression from sBC could protect them from immune recognition and destruction by CD8 T cells. HLA class I molecules are composed of 3 polymorphic alpha helixes and 1 invariable beta-2 microglobulin (B2M) chain (**Figure 2H**). B2M is common in all HLA class I isotypes and is required for proper localization of the HLA class I complex to the cell membrane. Thus, we employed CRISPR/Cas9 technology to target exon 1 and 2 of the invariable B2M gene in the iP line using specific guide RNAs (**Figure 2I**). Sanger sequencing further confirmed a thymine insertion and an exon 1-2 deletion modifications, effectively generating a B2M knock-out (KO) line, termed iP-BKO (**Supplementary Figure 2C**). To confirm that the clonal iP-BKO line displayed impaired HLA class I surface expression, WT and iP-BKO cells were subjected to IFN-*γ* (100ng/ml) stimulation for 48 hr. Flow cytometric and immunofluorescence staining confirmed the lack of HLA class I surface expression in iP-BKO cells (**Figures 2J, K**). Quantification of flow cytometric data indicates that around ~99% of all treated iP-BKO cells lack cell surface expression of HLA class I molecules (**Supplementary Figure 2D**). Taken together, these data confirm the generation of a B2M KO in iP cells resulting in a functional deletion of surface HLA class I expression.

### iP and iP-BKO Cell Lines Can Differentiate Into sBC With Similar Efficiencies

**Figure 3:**
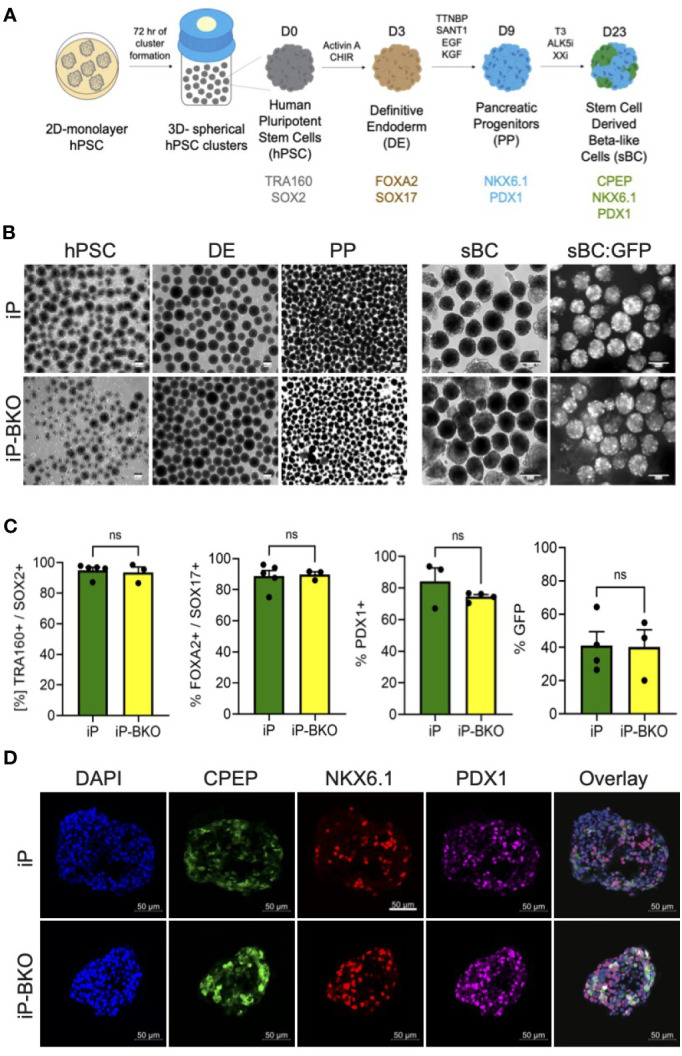
iP and iP-BKO hPSC lines differentiate into sBC with similar efficiencies. **(A)** Schematic of stepwise, suspension culture-based, direct differentiation approach for the generation of sBC clusters from hPSC. Critical induction molecules and gene markers at key differentiation stages are shown. **(B)** Representative bright field images of differentiating hPSC clusters in bioreactors at key developmental stages (left panel). Live images of insulin promoter-driven GFP expression at sBC stage (right panel). Note the higher magnification for sBC compared to earlier hPSC, DE and PE stages. All scale bars represent 200um. **(C)** Quantification of stage specific marker expression for iP and iP-BKO differentiating clusters using flow cytometric analysis. Data is presented as SEM +/- percentage of positive cells for indicated markers. Unpaired student t test was performed with no significant changes denoted as ns. **(D)** Representative immunofluorescence images of sBC derived from iP and iP-BKO cell lines. Samples were stain for DAPI (blue), CPEP (green), NKX6.1 (red), and PDX1 (purple) are shown. Scale bar represents 50um.

In order to test whether genetic modifications impair the ability of iP and iP-BKO hPSC lines to differentiate into sBC, both lines were subjected to a 3D beta cell differentiation protocol based on previous published work by us that generates glucose responsive sBCs (**Figure 3A**) (9, 10). Samples were taken at subsequent differentiation stages for morphological and flow cytometric analysis. No significant morphological differences were observed between the two cell lines at any stage throughout the entire differentiation protocol (**Figure 3B**). Flow cytometric analysis for pluripotency markers SOX2 and TRA160 showed that ~97% of cells stained positive for both markers after hPSC 3D cluster formation (**Figure 3C**).

Differentiation into the definitive endoderm lineage, marked by double expression of transcription factors Forkhead Box A2 (FOXA2) and SRY-Box 17 (SOX17), was highly efficient in both cell lines with >80% of all cells expressing both markers (**Figure 3C**). Subsequent differentiation into pancreatic progenitors, marked by the transcription factor Pancreatic and Duodenal Homeobox 1 (PDX1), was similarly efficient in both cell lines with >70% of all cells expressing PDX1 at this stage. Finally, sBC generation was monitored by GFP expression which is controlled by the endogenous insulin promoter. At the end of the differentiation protocol ~40% of all cells expressed GFP, indicating efficient generation of sBC. We did not find statistical differences between iP and iP-BKO cell lines at any stage of the sBC differentiation protocol (**Figure 3C**). Immunofluorescence analysis of sBC sections showed expression of key beta cell markers CPEP, NKX6.1 and PDX1 in both cell lines (**Figure 3D**). These data indicate that both iP and iP-BKO cells can differentiate into sBC at similar efficiencies, and that iP and iP-BKO genetic modifications do not alter differentiation potential into sBC.

### PD-L1 Overexpression and HLA Class I KO Abrogate Diabetogenic CD8 T Cell Activation

**Figure 4:**
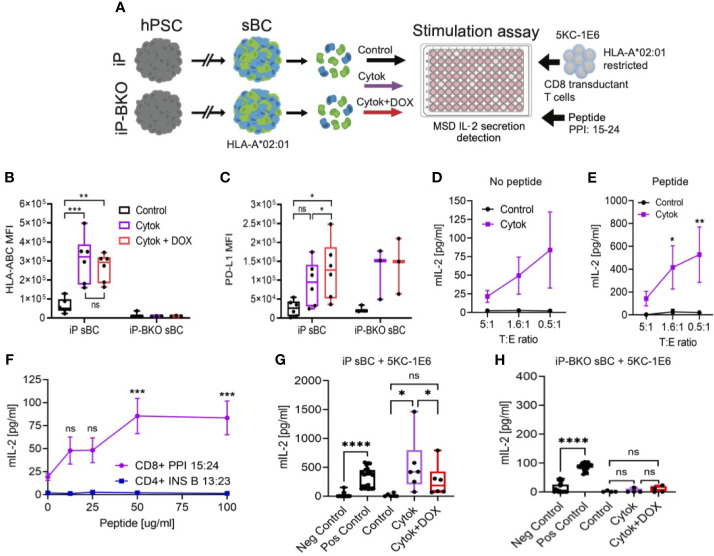
sBC activate a preproinsulin specific CD8 T cell receptor transductant in an HLA and PD-L1 dependent manner. **(A)** Schematic depicting the sBC and CD8 T cell receptor transductant (5KC-1E6) co-culture experimental assay set up. iP and iP-BKO cells were differentiated into sBC as clusters, followed by dissociation into single cells. sBC were treated with control (black), 2-day pro-inflammatory cytokines (purple) or with pro-inflammatory cytokines and DOX (red) for 48hr. sBC were then incubated with preproinsulin (PPI) 15-24 peptide for 4 hours. This was followed by 5KC-1E6 co-culture overnight with measurement of mIL-2 from the cell culture supernatants. **(B, C)** Flow cytometry quantification of surface HLA-ABC **(B)** or PD-L1 **(C)** expression on iP and iP-BKO sBC after treatments. Data is shown as MFI in box and whisker plots. Each dot represents a single differentiation experiment. Ordinary One-Way Anova was performed with Tukey’s multiple comparison test with *p ≤ 0.05, **p ≤ 0.01, ***p ≤ 0.001 and ns denoting no significant changes. **(D, E)** Stimulation assay of sBC treated with (purple) or without cytokines (black) followed by co-culture with 5KC-1E6 T cells at denoted target to effector rations (T:E). Note that sBC were cultured with **(E)** or without **(D)** exogenous PPI: 15-24 peptide. Data is presented as mean secreted mIL-2 concentration +/- SEM of 5 independent differentiation experiments. 2-way Anova with Sidak’s multiple comparison test was performed with *p ≤ 0.05, and **p ≤ 0.01. **(F)** Peptide titration experiment of different peptide-TCR combinations. sBC were treated with cytokines and incubated with different concentrations of PPI: 15-24 (purple line) or INS B 13:23 (blue line) peptides. sBC were co-culture with corresponding CD8 T cell receptor transductants. Data is presented as mean secreted mIL-2 concentration +/- SEM of 3 independent differentiation experiments. 2-way Anova with Sidak’s multiple comparison test was performed with ***p ≤ 0.001, and ns denoting no significant changes. **(G, H)** Stimulation assay of co-culture of 5KC-1E6 cells with iP **(G)** or iP-BKO **(H)** sBC after denoted treatments and incubated with exogenous PPI: 15-24. 5KC-1E6 alone or treated with anti-CD3 antibody served as negative and positive controls, respectively. Note the significant reduction in T cell stimulation upon PD-L1 expression (red) compared to only cytokine treatment (purple). Data is shown as box and whisker plots of secreted mIL-2. Each dot represents an independent stimulation assay with sBC from independent differentiation experiments. A paired t-test was performed with ns as no significant and *p ≤ 0.05, ****p ≤ 0.0001.

In order to test whether sBC will stimulate human diabetogenic CD 8 T cells, a reliable *in vitro* assay to measure T cell stimulation was developed. iP and iP-BKO hPSC were differentiated into sBC, followed by dissociation and plating on Matrigel coated 96 well plates to generate a 2D sBC monolayer for subsequent experiments. First, we exposed 2D sBC to a 2-day acute (IFN-*γ* 100ng/ml, IL-1β 5ng/ml and TNF-α 10ng/ml) cytokine treatment alone to upregulate HLA class I molecules, or to a 2-day acute cytokine treatment plus DOX to additionally induce PD-L1 expression (**Figure 4A**). Flow cytometric analysis revealed that iP sBC upregulated HLA class I molecules at similar levels in both conditions, while HLA class I surface expression was not detected on iP-BKO sBC (**Figure 4B**). PD-L1 surface expression was detected on 2D sBC from both cell lines after cytokine treatment, differing from 3D sBC cultures exposed to chronic cytokine treatment (**Figure 1E**). Moreover, adding DOX to the cytokine treated sBC further increased PD-L1 expression significantly (**Figure 4C**). Nevertheless, this data confirms that iP sBC can be induced to express HLA class I and PD-L1 surface proteins, while iP-BKO sBC can only express PD-L1.

Diabetogenic CD8 T cells are known to play an important role in beta cell destruction and diabetes progression (15). Preproinsulin (PPI) is a major self-antigen in T1D. CD8 T cells responding to PPI: 15-24 presented by HLA-A*02:01 have been shown to lyse human beta cells (27, 28). In order to test whether sBC can be targeted by a preproinsulin specific CD8 T cell, iP sBC were co-cultured with a T cell receptor (TCR) transductant expressing a TCR that responds to preproinsulin PPI: 15-24 peptide presented by HLA-A*02:01 (termed 5KC-1E6). The TCR α/β genes from the 1E6 T cell clone were retrovirally expressed in an immortalized T cell devoid of endogenous TCR expression, thus creating a TCR transductant that secretes interleukin-2 (IL-2) when stimulated with PP1: 15-24 presented by HLA-A*02:01 (29). Of note, the parental hPSC line Mel1, from which iP and iP-BKO lines were derived, contains the HLA-A*02:01 allele (**Supplementary Figure 3A**). T cell activation was measured by quantifying IL-2 secreted into the media after co-culture with sBC. Co-cultures of 5KC-1E6 T cells and iP sBC without cytokine treatment did not result in significant IL-2 secretion (**Figure 4D**). This is likely due to the low abundance of the PPI: 15-24 produced by sBC in culture and the low expression of HLA class I molecules at steady state. However, co-culture experiments with prior exposure of 2D sBC to pro-inflammatory cytokines resulted in a low but reproducible detection of IL-2 indicating activation of a PPI specific T cell with an inflammatory stimulus (**Figure 4D**). These results indicate that sBC can activate diabetogenic CD8 T cells in an inflammatory environment.

Next, similar experiments were repeated with the addition of exogenous PPI: 15-24 peptide to sBC cultures prior to co-culturing with 5KC-1E6 T cells. This resulted in a more robust, approximately 8-fold increased, secretion of IL-2 in cytokine treated sBC cultures (**Figure 4E**, **Supplementary Figures 3B, C**). As expected, sBC cultures not treated with cytokines did not trigger T cell stimulation despite the addition of exogenous PPI: 15-24 peptide (**Figure 4E**), indicating the importance and specificity of upregulating HLA class I molecules for CD8 T cell recognition. To determine if the IL-2 release in cytokine treated cultures was a specifically due to PPI: 15-24 bound to HLA-A*02:01 on sBC, a peptide titration was performed. There is a peptide binding curve for increased IL-2 with increasing PPI peptide concentration with a saturation level around 50ug/ml of PPI: 15-24 (**Figure 4F**). As a T cell specificity control, an insulin-specific CD4 TCR transductant (5KC-clone 5) that responds to insulin B chain amino acids 13-23 presented by the T1D risk HLA class II molecule, HLA-DQ8, was used in these experiments (30). 5KC-clone 5 was not activated to secrete IL-2 despite the presence of cytokines and exogeneous addition of cognate insulin peptide suggesting that activation of T cell is dependent on peptide presentation by sBC (**Figure 4F**). Taken together, these experiments indicate sBC can specifically activate a PPI reactive CD8 T cell in the presence of pro-inflammatory cytokines and the response is increased with the addition of PPI: 15-24 presented by HLA-A*02:01. Thus, sBC are susceptible to diabetogenic CD8 T cell mediated targeting in an HLA-peptide-TCR dependent manner.

Using this human sBC – CD8 T cell interaction model, whether PD-L1 overexpression and/or HLA KO reduces PPI CD8 T cell activation was investigated. iP and iP-BKO 2D sBC were treated with 2-days cytokines alone or 2-days cytokines plus DOX, followed by PPI: 15:24 peptide addition and co-culture with 5KC-1E6 T cells. As anticipated, iP sBC that were treated with cytokines stimulated T cells by releasing IL-2 (**Figure 4G**). However, T cell stimulation was significantly reduced when PD-L1 overexpression was induced with DOX (**Figure 4G**). Of note, 5KC-1E6 T cells do express mouse PD-1 protein on their surface (**Supplementary Figure 3D**). Experiments conducted using iP-BKO sBC (e.g. those lacking HLA class I molecules) showed no stimulation of 5KC-1E6 T cells even when exogenous PPI: 15-24 peptide was added (**Figure 4H**).

Collectively, this study and presents a novel model system assay that allows for detailed studies into human beta cell – T cell interaction. This system is also amenable to test genetic engineering models of immune protection. Finally, the protective role of PD-L1 in sBC was confirmed using this model system.

## Discussion

The use of hPSC for the generation of sBC could provide an unlimited source of functional beta-like cells for cell replacement therapy. However, the response of sBC to an inflammatory environment has not been well studied. Here we show that sBC upregulate HLA class I molecules upon pro-inflammatory cytokine exposure, similar to the response of cadaveric human islets to an inflammatory insult. Furthermore, HLA class I isotype specific gene expression analysis revealed that HLA-C is the predominant HLA class I molecule upregulated in sBC. This result differs from human islets where all HLA isotypes are upregulated at similar levels. It has been shown that HLA-C is the main isotype expressed in fetal trophoblasts (24, 25). It is intriguing to speculate that sBC, which have been proposed to closer resemble fetal rather than adult beta cells (31), might display an immature immune profile when challenged. However, further studies are warranted to clarify this possibility. Cadaveric beta cells have been shown to upregulate PD-L1 in response to pro-inflammatory cytokines (16–18) and our data confirms this result. However, we find that sBCs differ from cadaveric beta cells and do not express PD-L1 under the proinflammatory conditions employed in our study. This is in contrast to other reports that show PD-L1 expression on sBCs after treatment with high levels of proinflammatory cytokines (22). While further studies are required to clarify this point, we anticipate that regulation of gene expression of immune regulatory proteins in sBCs is highly context dependent.

Although sBC have been demonstrated to reverse diabetes in multiple mouse models, we studied their interaction with diabetogenic immune cells in a strictly human context. Co-culture of sBC and peripheral blood mononuclear cells derived from the same type 1 diabetic patient could represent a true autologous human system to study autoimmune diabetes *in vitro* (32). However, the low frequency of diabetogenic T cells found within the peripheral blood of T1D patients complicates the interpretation of these studies. Here, we provide an innovative platform to model HLA matched T cell – beta cell interactions for the study of autoimmune diabetes. Unlike peripheral blood immune cells, T cell receptor transductants represent an abundant and reusable source of antigen specific T cells (29, 33).

Here, we demonstrate that PPI reactive CD8 T cells transductants are stimulated by sBC in a TCR-peptide-HLA dependent manner when an inflammatory stimulus is present. This model system can be used for future studies to examine T cells of other specificities such as those responding to glutamic acid decarboxylase (GAD), insulinoma antigen-2 (IA-2), zinc transporter 8, hybrid insulin peptides and neoepitopes, and other immune cell subsets involved in T1D pathogenesis. This data supports previous studies in which immunogenicity of sBC to PPI: 15-24 specific cytotoxic T lymphocytes (CTLs) was described (34). Moreover, this study further demonstrates that genetic engineering of sBC could represent a viable strategy to avoid diabetogenic CD8 T cell activation. Future studies using diabetogenic CTLs co-culture with immune engineered sBCs could further substantiate this notion.

Manipulating HLA class I molecules using genetic engineering of hPSC that are differentiated into sBC could potentially provide an unlimited source of beta cells that are protected from a diabetogenic immune attack. Here, we knocked-out B2M which resulted in a functional KO for all HLA class I isotypes. hPSC that lack expression of HLA class I molecules successfully differentiated into sBC and did not stimulate PPI reactive T cells. We found that WT sBC exposed to an inflammatory environment upregulated HLA class I molecules and induce higher levels of activation of T cell transductants compared to sBC cultures at steady state with low levels of HLA class I expression. These results highlight the important role of cytokine-induced HLA class I molecules in immune cell activation and peptide presentation from stem cell derived cells. However, complete ablation of all HLA class I molecules has been shown to render cells vulnerable to a natural killer (NK) cell lysis (35, 36). Different alternative approaches to overcome NK cell targeting have been investigated. Targeted disruption of classical HLA-A, -B and -C while retaining expression of non-classical HLA-E alone (37) or by the combinatorial expression of HLA-G, CD47 and PD-L1 (38, 39) have been shown to protect hPSC from NK cell recognition and lysis. For these reasons, we explored the possibility of overexpressing the PD-L1 receptor and keeping expression of all HLA class I molecules to avoid NK cell lysis.

We successfully generated a knock-in of a Tet-On PD-L1 inducible expression system in the safe harbor locus AAVS1 in hPSC. This resulted in the induction of PD-L1 in ~95% of the hPSC. Our results further show that by overexpressing the immune checkpoint receptor PD-L1 in sBC, T cell stimulation is abrogated. A recent study showed that PD-L1 overexpression resulted in increased survival of sBC after 4 weeks of transplantation into the kidney capsule of immune competent and humanized mouse models (22). These studies showed increased survival and decreased immune infiltration of sBC transplants in a xenogeneic model system. Moreover, the specificity of immune – beta cell interface was not analyzed in this study. In contrast, our present study shows the direct interaction and activation of diabetogenic CD8 T cells by sBC and that PD-L1 expression can reduce T cell stimulation in an *in vitro* model of human T1D devoid of xenogeneic inputs.

In conclusion, we provide a reliable assay for the study of human sBC and diabetogenic T cell interactions *in vitro*. Using this assay, we further confirmed that sBC are vulnerable to diabetogenic T cell activation and that PD-L1 expression on sBC is sufficient to reduce T cell stimulation. Finally we provide a proof of principle that genetic engineering of human pluripotent stem cells can be used to generate immune protected stem cell derived beta-like cells that do not activate diabetogenic T cells.

## Data Availability Statement

The original contributions presented in the study are included in the article/**Supplementary Material**. Further inquiries can be directed to the corresponding author.

## Author Contributions

Conception and study design: RC-G, AM, and HR. Execution of experiments: RC-G, AA, and HR. Data analysis and interpretation: RC-G, AA, CM, AM, and HR. Manuscript writing: RC-G, AM, and HR. Final approval of manuscript: RC-G, AA, CM, AM, and HR. HR is the guarantor of this work and takes full responsibility for the work as a whole. All authors contributed to the article and approved the submitted version.

## Funding

This work was supported by NIH Grants (DK108868, DK032083, DK099317 and DK116073 to AM, DK104194, DK127497, DK122638 and AI042288 to CEM, and DK120444 and AI140044 to HAR, P30-DK116073 to University of Colorado Diabetes Research Center), a new investigator award from the NIDDK- supported Human Islets Research Network (HIRN, RRID : SCR_014393; UC24 DK1041162) (HR), a Culshaw Junior Investigator Award in Diabetes (HR), the Juvenile Diabetes Research Foundation (JDRF 2-SRA-2019-781-S-B) (HR), the Children’s Diabetes Foundation, and the Colorado Clinical and Translational Science Institute (TR002535 to AM). RC-G was supported by an RNA Bioscience Initiative Scholar award.

## Conflict of Interest

HAR is an islet biology SAB member at Sigilon therapeutics, consultant to Eli Lilly and SAB member at Prellis Biologics.

The remaining authors declare that the research was conducted in the absence of any commercial or financial relationships that could be construed as a potential conflict of interest.

## Supplementary Material

Supplementary Figure 1
**(A)** Representative immunofluorescence images of cluster sections from control and 6-day cytokine treated sBC stained for DAPI (blue), CPEP (green), HLA-ABC (purple), and PD-L1 (red). Scale bar represents 50um. **(B, C)** qPCR analysis of HLA class I isoforms and PD-L1 gene expression in control and 6-day cytokine treated human islets **(B)** and sBC **(C)**. Values are normalized to ACTB and results are shown as mean +/- SEM of n = 3 independent donors and experiments.

Supplementary Figure 2
**(A)** Schematic illustrating AAVS1 genomic region with Tet-On inducible PD-L1 system integration. Arrows delineate primers generated to test for correct integration of the inducible PD-L1 expression system (top panel). Agarose DNA gel showing genomic PCR amplification results from the AAVS1 region of WT and iP cells using indicated primer combinations (bottom panel). **(B)** Complete immunoblotting image of WT and iP cells with and without DOX treatment (2ug/ml for 48hr). PD-L1 band was detected at ~42kDa, while loading control GAPDH band was detected at ~37kDa as marked by protein standards. **(C)** Sanger sequencing results from bands obtained after genomic DNA amplification of the targeted B2M region. Green, yellow and blue boxes represent different DNA amplification bands that were extracted for Sanger sequencing analysis. Compared to WT genomic B2M gene sequencing (green box), iP-BKO B2M gene sequencing showed a thymine insertion (yellow box) on one allele and a deletion of ~3906 base pairs resulting in exon 1 and exon 2 fusion in the second allele (blue box) demonstrating two different genomic alterations in each of two clonal cell lines analyzed. **(D)** Flow cytometry histogram of surface HLA-ABC protein expression on WT and iP-BKO cells treated with IFN-*γ* (100ng/ml for 48hr). WT (pink) WT+IFN-*γ* (purple), iP-BKO (light green), iP-BKO+IFN-*γ* (dark green), unstained control (black) and isotype control (gray). A representative histogram normalized to mode from 3 independent experiments is presented.

Supplementary Figure 3
**(A)** Flow cytometry histogram of surface HLA-A2 on iP sBC after pro-inflammatory cytokine treatment (purple) compared to unstained sample (black). Data is presented in a representative histogram normalized to mode. **(B, C)** Stimulation assay of 5KC-1E6 PPI: 15-24 specific T cells after overnight co-culture at different T:E ratios with iP **(A)** or iP-BKO **(B)** sBC pre-treated with or without pro-inflammatory cytokines and with different concentrations of exogeneous peptide. Data is shown as mean of secreted mIL-2 +/- SEM of 5 independent experiments. 2-way Anova with Sidak’s multiple comparison test was performed in which *p ≤ 0.05, and ****p ≤ 0.0001. **(D)** Flow cytometry histogram of surface murine PD1 on 5KC-1E6 (PPI 15-24 specific) T cells (red) compared to unstained cells (gray). Data is presented in a representative histogram normalized with overall cell count.
