# Supplementary figures and images for: Protecting Stem Cell Derived Pancreatic Beta-Like Cells From Diabetogenic T Cell Recognition
Source: Front Endocrinol (Lausanne). 2021 Jul 9;12:707881. doi: 10.3389/fendo.2021.707881 (PMC8299417; doi:10.3389/fendo.2021.707881)

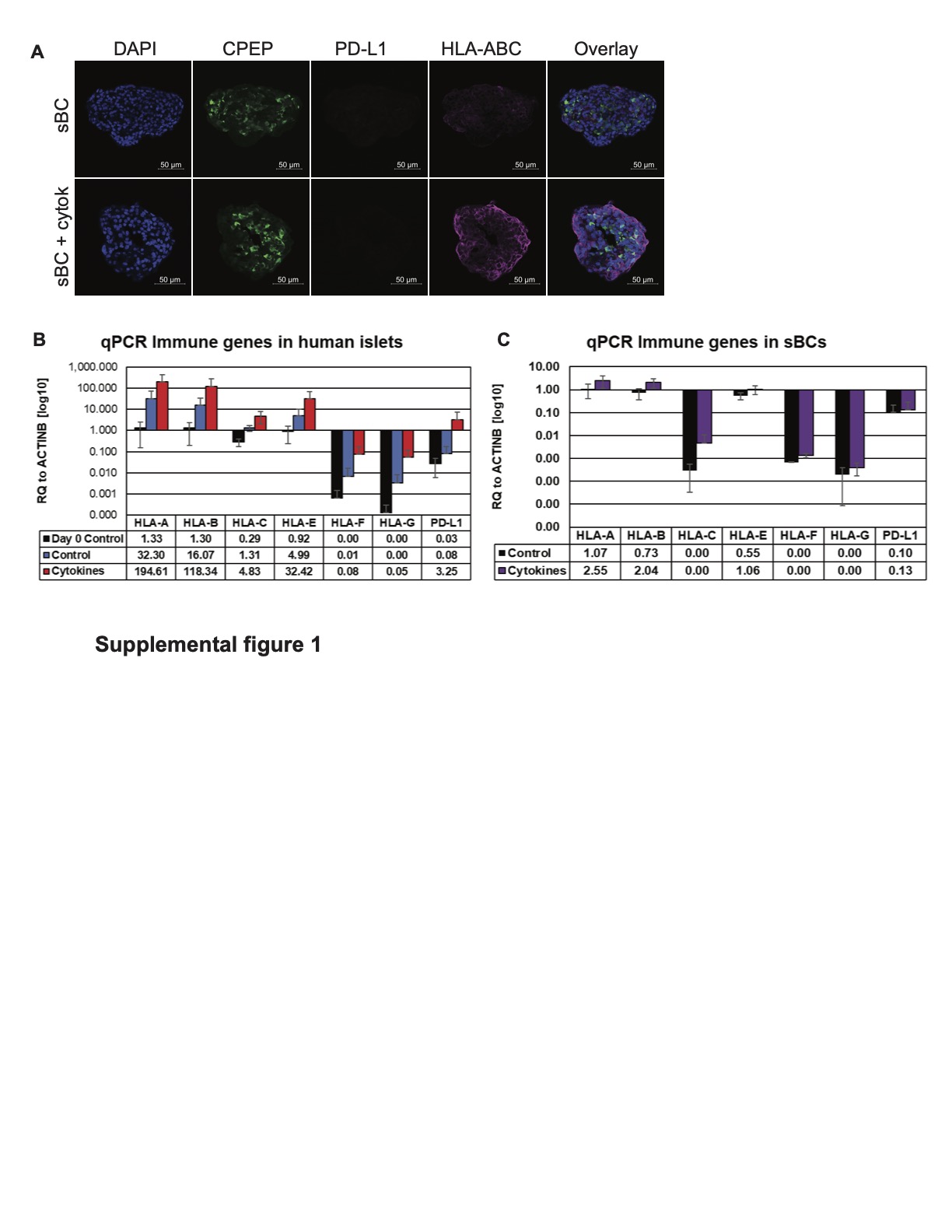

Supplement: Supplementary Figure 1 — (A) Representative immunofluorescence images of cluster sections from control and 6-day cytokine treated sBC stained for DAPI (blue), CPEP (green), HLA-ABC (purple), and PD-L1 (red). Scale bar represents 50um. (B, C) qPCR analysis of HLA class I isoforms and PD-L1 gene expression in control and 6-day cytokine treated human islets (B) and sBC (C). Values are normalized to ACTB and results are shown as mean +/- SEM of n = 3 independent donors and experiments. [file Image_1.jpeg]

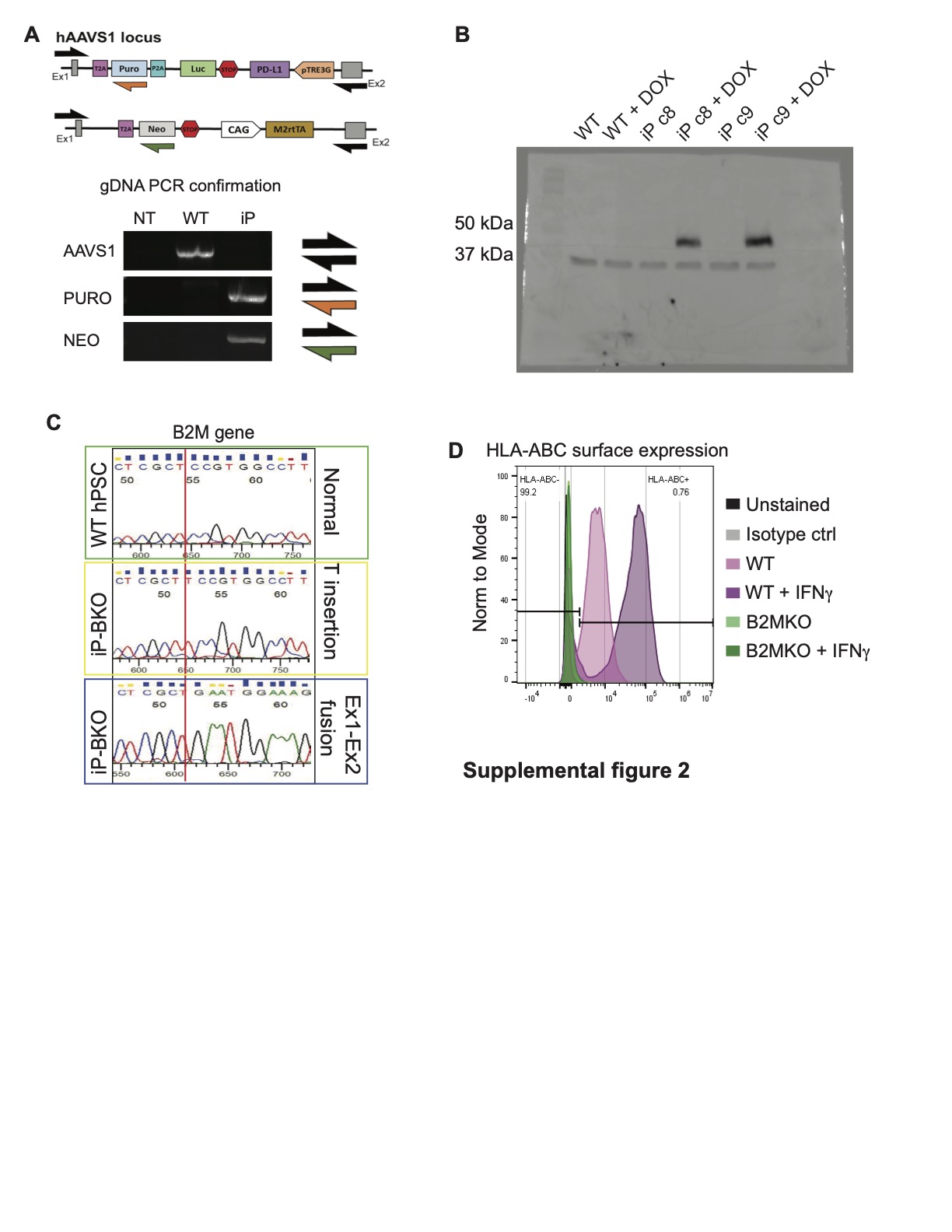

Supplement: Supplementary Figure 2 — (A) Schematic illustrating AAVS1 genomic region with Tet-On inducible PD-L1 system integration. Arrows delineate primers generated to test for correct integration of the inducible PD-L1 expression system (top panel). Agarose DNA gel showing genomic PCR amplification results from the AAVS1 region of WT and iP cells using indicated primer combinations (bottom panel). (B) Complete immunoblotting image of WT and iP cells with and without DOX treatment (2ug/ml for 48hr). PD-L1 band was detected at ~42kDa, while loading control GAPDH band was detected at ~37kDa as marked by protein standards. (C) Sanger sequencing results from bands obtained after genomic DNA amplification of the targeted B2M region. Green, yellow and blue boxes represent different DNA amplification bands that were extracted for Sanger sequencing analysis. Compared to WT genomic B2M gene sequencing (green box), iP-BKO B2M gene sequencing showed a thymine insertion (yellow box) on one allele and a deletion of ~3906 base pairs resulting in exon 1 and exon 2 fusion in the second allele (blue box) demonstrating two different genomic alterations in each of two clonal cell lines analyzed. (D) Flow cytometry histogram of surface HLA-ABC protein expression on WT and iP-BKO cells treated with IFN-γ (100ng/ml for 48hr). WT (pink) WT+IFN-γ (purple), iP-BKO (light green), iP-BKO+IFN-γ (dark green), unstained control (black) and isotype control (gray). A representative histogram normalized to mode from 3 independent experiments is presented. [file Image_2.jpeg]

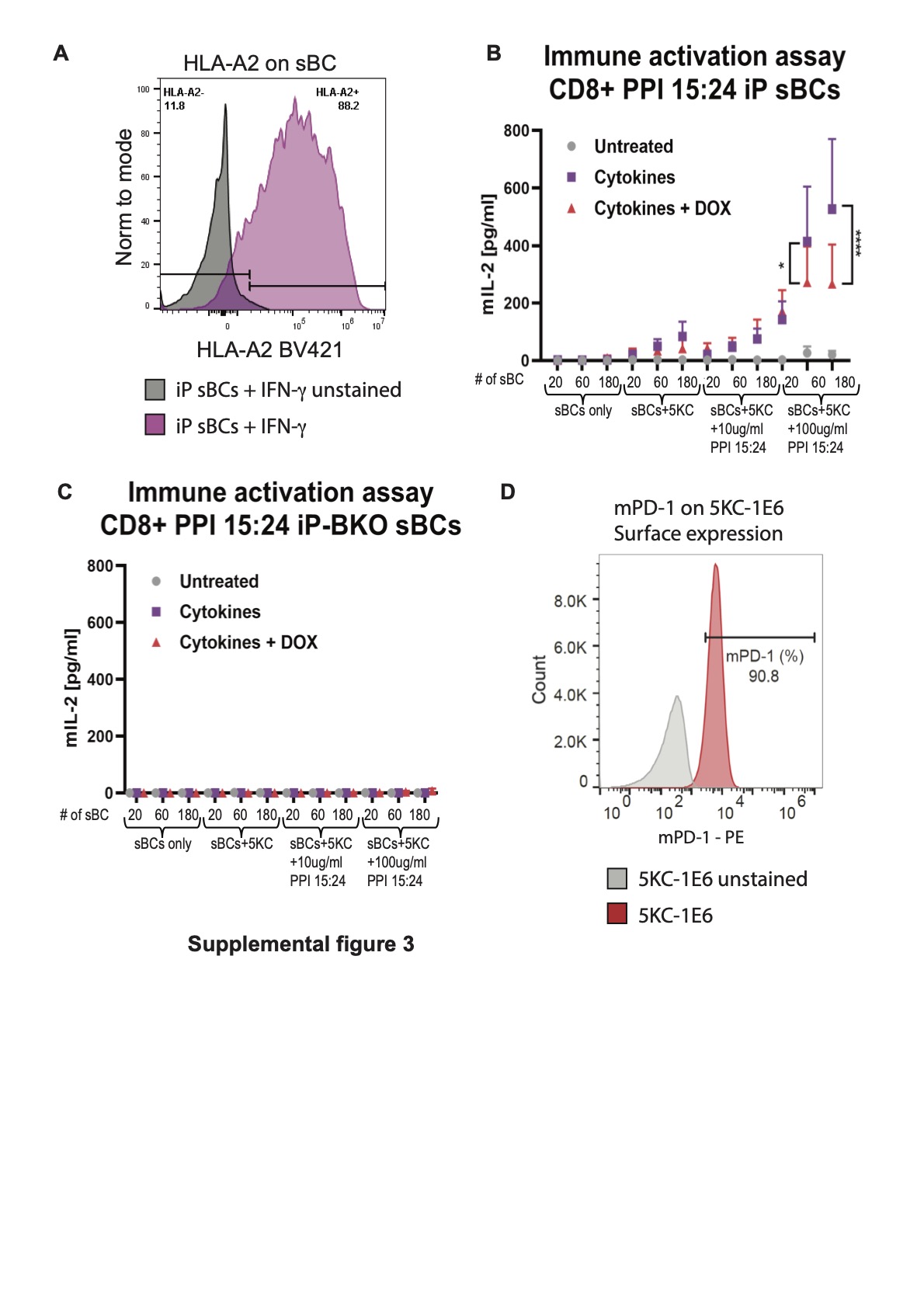

Supplement: Supplementary Figure 3 — (A) Flow cytometry histogram of surface HLA-A2 on iP sBC after pro-inflammatory cytokine treatment (purple) compared to unstained sample (black). Data is presented in a representative histogram normalized to mode. (B, C) Stimulation assay of 5KC-1E6 PPI: 15-24 specific T cells after overnight co-culture at different T:E ratios with iP (A) or iP-BKO (B) sBC pre-treated with or without pro-inflammatory cytokines and with different concentrations of exogeneous peptide. Data is shown as mean of secreted mIL-2 +/- SEM of 5 independent experiments. 2-way Anova with Sidak’s multiple comparison test was performed in which *p ≤ 0.05, and ****p ≤ 0.0001. (D) Flow cytometry histogram of surface murine PD1 on 5KC-1E6 (PPI 15-24 specific) T cells (red) compared to unstained cells (gray). Data is presented in a representative histogram normalized with overall cell count. [file Image_3.jpeg]
